# Effect of Zirconium Oxide Nanofiller and Dibutyl Phthalate Plasticizer on Ionic Conductivity and Optical Properties of Solid Polymer Electrolyte

**DOI:** 10.1155/2014/547076

**Published:** 2014-07-15

**Authors:** Siti Mariah Mohd Yasin, Suriani Ibrahim, Mohd Rafie Johan

**Affiliations:** Nanomaterials Engineering Research Group, Advanced Materials Research Laboratory, Department of Mechanical Engineering, University of Malaya, Lembah Pantai, 50603 Kuala Lumpur, Malaysia

## Abstract

New solid polymer electrolytes (SPE) based on poly(ethylene oxide) (PEO) doped with lithium trifluoromethanesulfonate (LiCF_3_SO_3_), dibutyl phthalate (DBP) plasticizer, and zirconium oxide (ZrO_2_) nanoparticles were prepared by solution-casting technique. The conductivity was enhanced by addition of dibutyl phthalate (DBP) plasticizer and ZrO_2_ nanofiller with maximum conductivity (1.38 × 10^−4^ Scm^−1^). The absorption edge and band gap values showed decreases upon addition of LiSO_3_CF_3_, DBP, and ZrO_2_ due to the formation of localized states in the SPE and the degree of disorder in the films increased.

## 1. Introduction

Nowadays, the need for smaller, lighter, higher capacity, and energy density devices are tremendous especially in the field of polymer electrolytes materials. Extensive studies were conducted on solid polymer electrolytes (SPE) as new ionic conductors to replace the conventional electrolytic solutions [1]. It is believed that the electrolytic solutions have solution leakage, electrode corrosion, contamination, and solute seeping.

SPE based on poly(ethylene oxide) (PEO) have received much attention due to its well-dissolved salts and such electrolytes possess proper chemical structures to support the ion transport [2]. Many researchers discovered that these polymer electrolytes have low conductivities [3]. Nevertheless, they realized that polymer electrolytes have a big potential for future technology development. Since then, more investigations had been carried out to overcome the problem like the reduction of the PEO crystalline phase which is known to be contributed to its lower conductivity [4]. In that regard, addition of low molecular weight plasticizer into the polymer electrolytes systems will enhance the conductivity [5].

The plasticization is the conventional way to reduce the crystallinity and enhances the amorphous phase content of the polymer electrolytes. Thus, it increases the flexibility in the polymeric segments and produces mobile charge carriers due to ion dissolution effect. In this work, dibutyl phthalate (DBP) was used as a plasticizer. This leads to high ambient conductivity. DBP plasticizer contributed higher ionic conductivity compared to ethylene carbonate (EC) and polycarbonate (PC) plasticizers. For example at room temperature, electrolyte system with DBP [6] as a plasticizer contributes to 10^−4^ S/cm compared to EC/PC [7, 8] with 10^−6^ S/cm.

However the conductivity enhancement occurred at the expense of SPE mechanical properties. To overcome this, the addition of nanosize ceramic fillers into the SPE system results in the increase of ionic conductivity coupled with its mechanical properties [9]. In this work, zirconium oxide (ZrO_2_) nanoparticles were used as ceramic filler into the host polymer matrix [10, 11]. The conductivity increases with decrease in particle size (nanometer), that is, increase in the ratio of surface area to volume, thus leading to an increasing dominance of the atomic behavior on the surface of particle compared to the dominance of the atomic behaviors in the interior of the particle. The larger surface area prevents the local polymer chain reorganization, leading to locking in high degree of disorder and enhancing the ionic conductivity. Moreover, the large surface area of ZrO_2_ nanoparticle results in a lot of interactions between the intermixed materials leading to special properties such as strength and increased chemical or heat resistance. Moreover, ZrO_2_ is the strongest and toughest ceramic material available for use in research and industrial area. It also offers potential for long-term stability. Filler will affect the polymer dipole orientation by their ability to align dipole moments. Addition of filler increases the ionic conductivity and mechanical stability of polymer electrolytes by inhibiting the recrystallization of polymer chains and providing Li^+^ conducting pathways at the filler surface through Lewis acid-base interaction among different species in the electrolyte. The ion movement is obstructed by the crystalline region present in polymer electrolyte while blocking the paths of the ions. The amorphous region favors the conduction of Li^+^ due to its greater free volume.

Optical study has been one of the most productive methods in understanding the band structure and energy gap of both crystalline and amorphous materials. In this light, the band structure of SPE will get affected due to the decrease of crystallinity as a result of adding plasticizer and filler. Studies on the ionic conduction is focusing on the nature of the charge transport that occurred in the polymer electrolyte while the optical properties are aimed at achieving better reflection, antireflection, interface, and polarization properties. The optical properties of polymers can be suitably modified by the addition of dopants depending on their reactivity with the host matrix. In our case, the dopants include the salt, plasticizer, and filler. We found that the doping elements are responsible for the formation of defects in the electrolyte film. By adding different element to the PEO complexes, optical band gap decreases for both direct and indirect transitions due to the compositional change in the host material. The decrease in the optical band gap results in an increase in the degree of disorder in the electrolyte films. As a result, the ionic conductivity of the film will be increased due to the increase of amorphous phase of the film. Therefore, ionic conductivity has a direct relationship with its optical property through their band gap energy.

Thus, the study on the effect of plasticizer and ceramic filler on PEO/salt based polymer electrolytes would be of great interest.

## 2. Experiment

Polymer electrolyte films were prepared using solution-cast technique. Polyethylene oxide (PEO, Aldrich) was used as host polymer matrix, lithium trifluoromethanesulfonate (LiCF_3_SO_3_, Aldrich) as the salt for complexation, dibutyl phthalate (DBP, Alfa Aesar) as plasticizer and zirconium oxide (ZrO_2_, Acros Organics) as nanoceramic filler. Prior to the preparation of polymer electrolyte, LiCF_3_SO_3_ was dried at 100°C for 1 h in order to eliminate trace amounts of water. 1 g of PEO (per mixture) and various wt.% of LiCF_3_SO_3_ were dissolved separately in acetonitrile (Fisher) and these solutions were then mixed together and stirred. For the second system, different amounts of plasticizer (DBP) were added and stirred with the amount of polymer and salt being fixed. Finally, different amounts of ceramic filler (ZrO_2_) were added and stirred with the amount of polymer, salt, and plasticizer being fixed. The solutions were stirred for 24 h and then were cast on petri dish and allowed to evaporate slowly inside a dessicator for about 4 days. All samples were stored under dry conditions.

Conductivity measurements were carried out using impedance spectroscopy HIOKI 3532 LCR Hi Tester, within the frequency range of 50 Hz to 1 MHz, with temperature ranging from room temperature to 373 K. A specimen's area for all testing was 3.142 cm^2^ and the thickness range within 0.010–0.020 cm. Optical studies were carried out using Cary 50 probe UV-visible spectrophotometer. The absorption spectra were analysed in a wavelength range of 190–800 nm.

## 3. Result and Discussion

### 3.1. Ionic Conductivity Study

**Figure 1:**
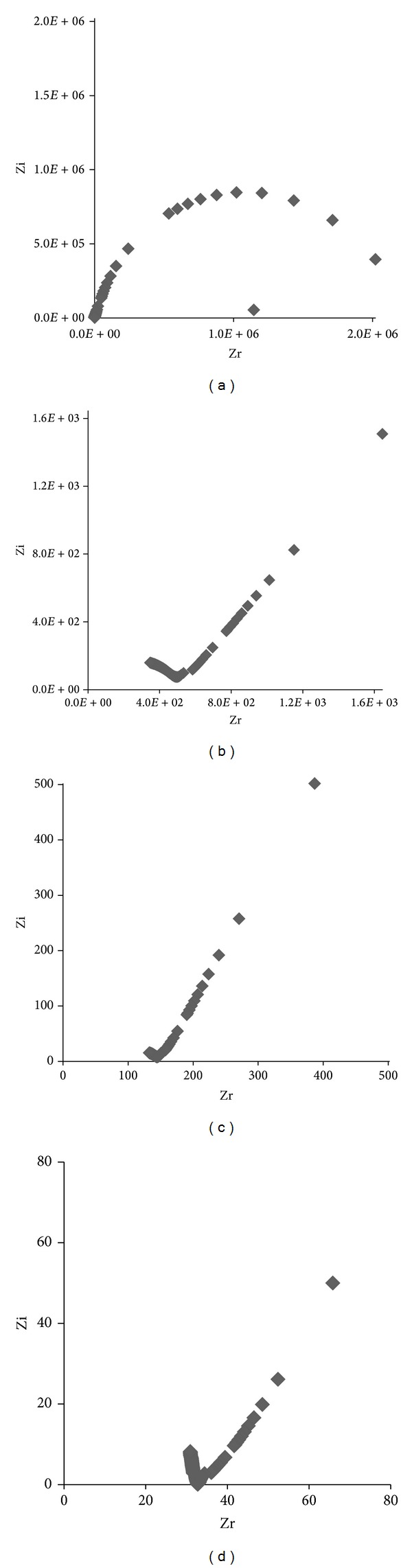
Cole-Cole plot for SPE samples: (a) PEO; (b) (PEO/LiCF_3_SO_3_); (c) (PEO/LiCF_3_SO_3_/DBP); (d) (PEO/LiCF_3_SO_3_/DBP/ZrO_2_) at ambient temperature.

Figure 1 shows the typical impedance plot of pure PEO, (PEO/LiCF_3_SO_3_), (PEO/LiCF_3_SO_3_/DBP), and (PEO/LiCF_3_SO_3_/DBP/ZrO_2_) at room temperature in the frequency range of 50 Hz to 5 MHz. The Cole-Cole plots of the films are consisting of a semicircular arc and inclined spike, both representing behaviour of ionically conducting solid electrolyte films with blocking electrode [12].

Figure 1(a) shows the semicircle arc due to the parallel combination of bulk resistance and bulk capacitance. The bulk resistance is due to the migration of ion and the bulk capacitance is due to the immobile polymer chains [13]. Figures 1(b)–1(d) show the inclination of the spike that have an angle less than 90° to the real axis due to the roughness of the electrode-electrolyte interface [14]. The inclined spikes indicate the double layer capacitance formed at the electrode-electrolyte interface because of ionic migration at low frequency. When the frequency decreases, the impedance against ion transfer increases, which is represented by the electrode double layer at each interface [15].

The ionic conductivity of the sample can be obtained using the equation below:

(1) σ=tRbA,

where *σ* is the conductivity, *t* (cm) is the thickness of the sample, *R*_*b*_ (Ω) is the bulk resistance, and *A* (cm^2^) is the area of the electrode and electrolyte contact. The *R*_*b*_ value is obtained from the intercept of Cole-Cole plot with *x*-axis.  Table 1 summarized the conductivity values of SPE calculated using (1). It shows that sample with 0.05 wt.% ZrO_2_ possesses the highest ionic conductivity.

**Figure 2:**
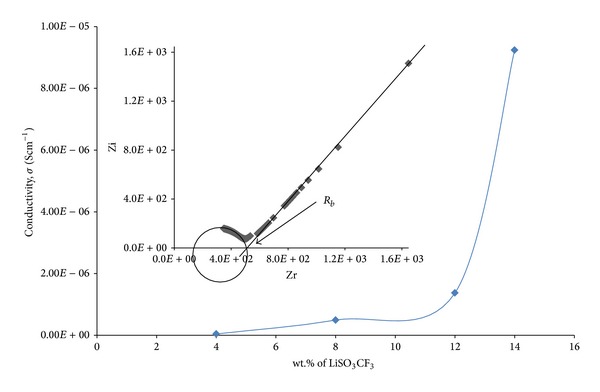
Ionic conductivities of SPE for various wt.% of LiSO_3_CF_3_ and impedance plot for sample with the highest conductivity (inset).

Figure 2 shows the conductivity of PEO + LiSO_3_CF_3_ with different wt.% of LiSO_3_CF_3_. The amount of salt was varied from 4 to 16 wt.% of LiSO_3_CF_3_. The highest conductivity of PEO + LiSO_3_CF_3_ system was obtained at 14 wt.% of LiCF_3_SO_3_, which is 9.24 × 10^−6^ Scm^−1^. Beyond 14 wt.% of LiSO_3_CF_3_, the sample was sticky and difficult to pry out from the petri dish. Hence, the sample failed to form film and remained permanently in a gel-like state. The inset in Figure 2 shows that intercept on the *Z*_*r*_ axis was attributed to the bulk resistance.

It is evident from Figure 2 that for different electrolyte films with various concentration of LiCF_3_SO_3_, their ionic conductivity increases to higher value with increasing of lithium ions due to the increase of charge carriers to the polymer electrolyte [16]. Besides that, the increase of conductivity is also attributed to the reduction in crystallinity of polymer electrolyte. The motion of lithium ions in this solid electrolyte is a liquid-like property. Therefore, the ionic movement through the SPE is facilitated by the large amplitude of the polymer segmental motion [17].

**Figure 3:**
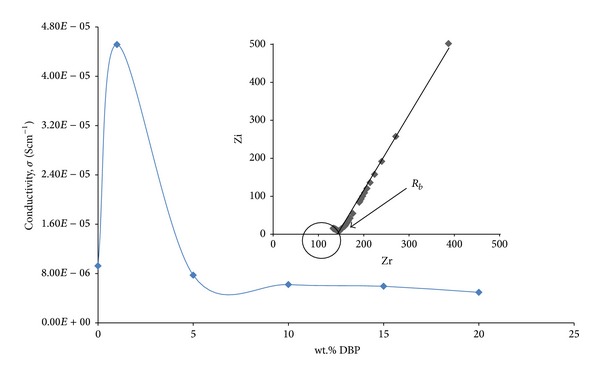
Ionic conductivities of SPE for various wt.% of DBP and impedance plot for sample with the highest conductivity (inset).

Figure 3 shows the variation in conductivity for PEO + LiSO_3_CF_3_ with different wt.% of DBP. The conductivity showed a maximum value at 1.0 wt.% of DBP (4.51 × 10^−5^ Scm^−1^) as shown in the inset of the figure. DBP appears to play a catalytic role in dissociating the salt and increasing the carrier concentration [3]. DBP increased the dissociation of LiCF_3_SO_3_ into Li^+^ and CF_3_SO_3_^−^ ions up to 1.0 wt.% of DBP. The value of conductivity decreases beyond 1.0 wt.% of DBP due to the diluents effect of DBP which lowers the number of Li^+^ ions per unit volume [1]. This could be ascribed to ion aggregation and, as a result, decreases the available charge carriers. The films also became sticky in nature [18].

**Figure 4:**
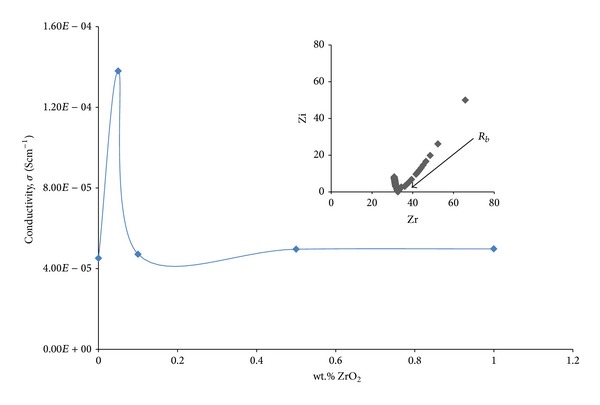
Ionic conductivities of SPE for various wt.% of ZrO_2_ nanofiller and impedance plot for sample with the highest conductivity (inset).

The Cole-Cole plot for the highest conductivity of PEO + LiCF_3_SO_3_ + DBP + ZrO_2_ film is shown (inset) in Figure 4. It is observed that the conductivity in filler system is higher than that in other systems. The maximum conductivity (1.38 × 10^−4^ Scm^−1^) was obtained for samples PEO + 14 wt.% LiCF_3_SO_3_ + 1 wt.% DBP + 0.05 wt.% ZrO_2_. However, the ionic conductivity does not continually rise indefinitely and declines beyond 0.05 wt.% of ZrO_2_. This could be ascribed to the fact that filler grains get close enough to each other as the filler concentration is further increased. The blocking effect or geometrical constrictions imposed by more abundant zirconia grains leading to long polymer chains become more “immobilized.” Consequently, the conductivity drops after reaching the maximum [19]. The addition of lower wt.% of filler into the SPE system slightly enhances the conductivity. This promotes structural modification of the polymer chains and thereby increases the fraction of the amorphous phase present at lower temperature [20]. Consequently, this favours Li^+^ ion migration. The mechanism of conductivity enhancement in SPE due to ceramic additives remains uncertain until present, although several models have been discovered [21]. However, the discovered mechanism is that ceramic additives reduce the host polymer crystallinity which, in turn, enhances the conductivity.

### 3.2. Optical Study

**Figure 5:**
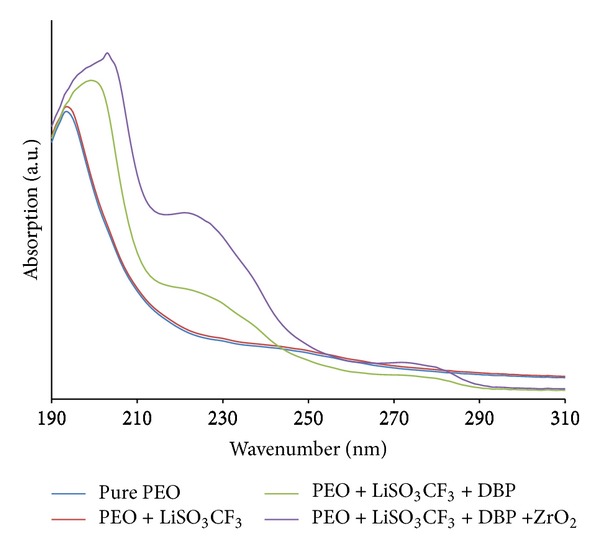
Optical absorption spectra for SPE for various wt.% of ZrO_2_ nanofiller.

Figure 5 shows the absorption spectra for pure and salted SPE around 197–204 nm. The bands exhibit different intensities and are assigned to the *n* → σ* transition from nonbonding to sigma antibonding orbitals. This transition usually comes from saturated compound containing atoms with nonbonding orbital, that is, a lone pair on oxygen, such as C–O bond in the polymer chain, whereas, the plasticized and filler added SPE were observed at around 218–237 nm. These may be attributed to the *π* → π*, which is the promotion of an electron from pi bonding orbital to pi antibonding orbital. The small peaks at around 270–289 nm were observed for both plasticized and filler systems can be assigned to the *n* → π*, which is promotion of an electron from nonbonding orbital to pi antibonding orbital. These transitions for both electrolyte systems need an unsaturated group in the molecule to provide the p electrons for the C=O bond in the plasticizer and filler molecules [22].

**Figure 6:**
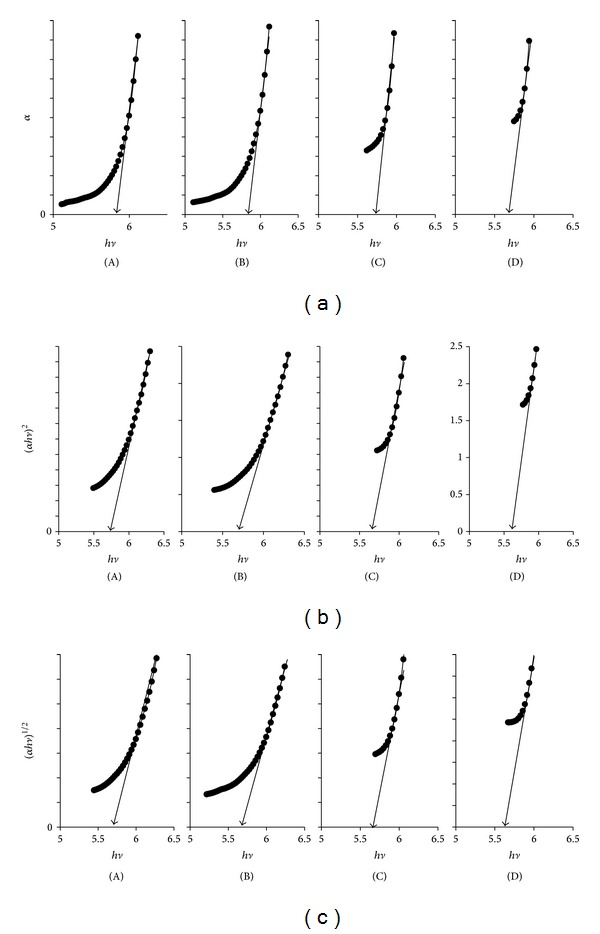
(a) Plot of *α* versus *hυ* (photon energy); (b) plot of (*α* *hυ*)^2^ versus *hυ* (direct band gap); (c) plot of (*α* *hυ*)^1/2^ versus *hυ* (indirect band gap) for (A) pure PEO; (B) PEO and LiSO_3_CF_3_; (C) PEO and LiSO_3_CF_3_ and DBP; (D) PEO and LiSO_3_CF_3_ and DBP + ZrO_2_.

Figure 6 shows the optical band gap of SPE (maximum conductivity) based on the Tauc plot equation for direct and indirect transitions [23]:

(2) $$\begin{array}{c} \left(\alpha h\upsilon \right)=\beta_{2}{\left(h\upsilon -E_{\text{gd}}\right)}^{2} \end{array}$$

(3) $$\begin{array}{c} \left(\alpha h\upsilon \right)=\beta_{1}{\left(h\upsilon -E_{\text{gi}}\right)}^{1/2}. \end{array}$$

Here *hυ* is the photon energy, *E*_gd_ is the direct band gap, *E*_gi_ is the indirect band gap, *α* represents the absorption coefficient, and *β*_1_ and *β*_2_ are the constants equal to (4*πσ*_*o*_/*nc*Δ*E*) whereby *n* is the refractive index.

The absorption edge values were obtained by extrapolating the linear portions of *α* versus *hυ* curves to zero absorption values, as shown in Figure 6(a). When a direct band gap exists, the absorption coefficient of electron transition depends on the energy of incident photon according to (2).

The direct band gap values were obtained from the plots of (*α* *hυ*)^2^ versus *hυ* and the allowed direct transition energies were determined by the intercept on the energy axis, on extrapolating the linear portion of the curves to zero absorption value as shown in Figure 6(b). For indirect electron transitions (*α* *hυ*)^1/2^ versus *hυ* as shown in Figure 6(c) which require photon assistance, the absorption coefficient has the following dependence on photon energy according to (3).

The values of absorption edge, direct band gap, and indirect band gap are shown in Table 2. It is clear that all values showed a decrease upon doping with salt LiSO_3_CF_3_, plasticizer DBP, and filler ZrO_2_. They are responsible for the defect formation in the electrolytes which produced the localized states in the optical band gap. Thus, this state was responsible for decreasing energy band gap.

## 4. Conclusion

Nanocomposite SPE have been successfully prepared by solution casting technique. The incorporation of LiCF_3_SO_3_ salt, DBP plasticizer, and ZrO_2_ nanofiller has led to significantly enhanced ionic conductivities. The composition of PEO + LiCF_3_SO_3_ + DBP + 0.05 wt.% ZrO_2_ exhibits the highest conductivity, (1.38 × 10^−4^ Scm^−1^), compared with the conductivity of pure PEO (1.58 × 10^−9^ Scm^−1^) and PEO + LiCF_3_SO_3_ (9.24 × 10^−6^ Scm^−1^). As the salt (LiSO_3_CF_3_) content increases, the conductivity increases because the density of mobile ions increases and, therefore, the polymer segment's motion is promoted. The incorporation of plasticizer (DBP) into the polymer electrolytes increases 4 orders of magnitude. It generally results from the reduction of crystallinity, the increase of salt dissociation capability, and the rise of charge carrier diffusions. However, the increase of conductivity of polymer electrolyte due to the plasticizer is coupled with a decrease in its mechanical strength. The incorporation of ceramic nanofiller seems to be an alternative provider of higher conductivity, cationic transference number, and mechanical strength. These three elements, salt, plasticizer, and nanosize filler, are mutually combined and connect towards formation of the superior solid polymer electrolyte. Each element (salt, plasticizer, and filler) is important and plays its specific role towards the formation of polymer electrolyte. It is very difficult to choose which one is more important since each contributes in their own way. Optical analysis of absorption edge, direct band gap, and indirect band gap values showed a decrease upon doping with LiSO_3_CF_3_, DBP, and ZrO_2_ to the pure PEO film. This incorporation was responsible for the formation of defects in the electrolytes. This produced the localized states in the SPE and the degree of disorder in the films increased.

## Figures and Tables

**Table 1:** Conductivity values of SPE at ambient temperature.

Sample	Conductivity *σ*
	(Scm^−1^)
PEO	1.58 × 10^−9^
PEO + 4 wt.% LiCF_3_SO_3_	4.17 × 10^−8^
PEO + 8 wt.% LiCF_3_SO_3_	4.91 × 10^−7^
PEO + 12 wt.% LiCF_3_SO_3_	1.37 × 10^−6^
PEO + 14 wt.% LiCF_3_SO_3_	9.24 × 10^−6^
PEO + 14 wt.% LiCF_3_SO_3_ + 1 wt.% DBP	4.51 × 10^−5^
PEO + 14 wt.% LiCF_3_SO_3_ + 5 wt.% DBP	4.96 × 10^−6^
PEO + 14 wt.% LiCF_3_SO_3_ + 10 wt.% DBP	5.94 × 10^−6^
PEO + 14 wt.% LiCF_3_SO_3_ + 15 wt.% DBP	6.20 × 10^−6^
PEO + 14 wt.% LiCF_3_SO_3_ + 20 wt.% DBP	7.73 × 10^−6^
PEO + 14 wt.% LiCF_3_SO_3_ + 1 wt.% DBP	1.38 × 10^−4^
+ 0.05 wt.% ZrO_2_	
PEO + 14 wt.% LiCF_3_SO_3_ + 1 wt.% DBP	4.71 × 10^−5^
+ 0.10 wt.% ZrO_2_	
PEO + 14 wt.% LiCF_3_SO_3_ + 1 wt.% DBP	4.96 × 10^−5^
+ 0.50 wt.% ZrO_2_	
PEO + 14 wt.% LiCF_3_SO_3_ + 1 wt.% DBP	4.98 × 10^−5^
+ 1.00 wt.% ZrO_2_	

**Table 2:** Absorption edge and band gaps values for SPE.

Polymer electrolyte	Absorption edge (eV)	Band gap (eV)	
		Direct	Indirect
Pure PEO	5.80	5.70	5.68
PEO + 14 wt.% LiCF_3_SO_3_	5.78	5.68	5.65
PEO + 14 wt.% LiCF_3_SO_3_	5.70	5.65	5.53
+ 1 wt.% DBP			
PEO + 14 wt.% LiCF_3_SO_3_	5.68	5.60	5.57
+ 1 wt.% DBP + 0.05 wt.% ZrO_2_			
